# Developing Cognition Endpoints for the CENTER-TBI Neuropsychological Test Battery

**DOI:** 10.3389/fneur.2020.00670

**Published:** 2020-07-17

**Authors:** Jonas Stenberg, Justin E. Karr, Douglas P. Terry, Simen B. Saksvik, Anne Vik, Toril Skandsen, Noah D. Silverberg, Grant L. Iverson

**Affiliations:** ^1^Department of Neuromedicine and Movement Science, Norwegian University of Science and Technology (NTNU), Trondheim, Norway; ^2^Department of Neurosurgery, St. Olavs Hospital, Trondheim University Hospital, Trondheim, Norway; ^3^Department of Physical Medicine and Rehabilitation, Harvard Medical School, Boston, MA, United States; ^4^Department of Psychiatry, Harvard Medical School, Boston, MA, United States; ^5^Spaulding Rehabilitation Hospital, Charlestown, MA, United States; ^6^Home Base, A Red Sox Foundation and Massachusetts General Hospital Program, Charlestown, MA, United States; ^7^Spaulding Research Institute, Charlestown, MA, United States; ^8^Department of Psychology, Norwegian University of Science and Technology (NTNU), Trondheim, Norway; ^9^Department of Physical Medicine and Rehabilitation, St. Olavs Hospital, Trondheim University Hospital, Trondheim, Norway; ^10^Department of Psychology, University of British Columbia, Vancouver, BC, Canada; ^11^Division of Physical Medicine & Rehabilitation, University of British Columbia, Vancouver, BC, Canada; ^12^Rehabilitation Research Program, GF Strong Rehabilitation Centre, Vancouver, BC, Canada

**Keywords:** brain concussion, brain injury, cognition, neuropsychology, psychometrics

## Abstract

**Background:** Measuring cognitive functioning is common in traumatic brain injury (TBI) research, but no universally accepted method for combining several neuropsychological test scores into composite, or summary, scores exists. This study examined several possible composite scores for the test battery used in the large-scale study Collaborative European NeuroTrauma Effectiveness Research in Traumatic Brain Injury (CENTER-TBI).

**Methods:** Participants with mild traumatic brain injury (MTBI; *n* = 140), orthopedic trauma (*n* = 72), and healthy community controls (*n* = 70) from the Trondheim MTBI follow-up study completed the CENTER-TBI test battery at 2 weeks after injury, which includes both traditional paper-and-pencil tests and tests from the Cambridge Neuropsychological Test Automated Battery (CANTAB). Seven composite scores were calculated for the paper and pencil tests, the CANTAB tests, and all tests combined (i.e., 21 composites): the overall test battery mean (OTBM); global deficit score (GDS); neuropsychological deficit score-weighted (NDS-W); low score composite (LSC); and the number of scores ≤5th percentile, ≤16th percentile, or <50th percentile.

**Results:** The OTBM and the number of scores <50th percentile composites had distributional characteristics approaching a normal distribution. The other composites were in general highly skewed and zero-inflated. When the MTBI group, the trauma control group, and the community control group were compared, effect sizes were negligible to small for all composites. Subgroups with vs. without loss of consciousness at the time of injury did not differ on the composite scores and neither did subgroups with complicated vs. uncomplicated MTBIs. Intercorrelations were high *within* the paper-and-pencil composites, the CANTAB composites, and the combined composites and lower *between* the paper-and-pencil composites and the CANTAB composites.

**Conclusion:** None of the composites revealed significant differences between participants with MTBI and the two control groups. Some of the composite scores were highly correlated and may be redundant. Additional research on patients with moderate to severe TBIs is needed to determine which scores are most appropriate for TBI clinical trials.

## Introduction

The European Commission has funded a large-scale, multi-national longitudinal observational study called the Collaborative European NeuroTrauma Effectiveness Research in Traumatic Brain Injury (CENTER-TBI) (1–4). Collectively, CENTER-TBI aspires to identify best practices, develop precision medicine, and improve outcomes for people with TBIs via comparative-effectiveness studies. Repositories of comprehensive clinical patient data, neuroimaging, genetics, and blood biomarkers are being developed that can be used to advance the field of brain injury medicine in diverse ways, including improving diagnosis, clinical management, and prognostication (1). The cognitive test battery used in CENTER-TBI includes both computerized and traditional paper-and-pencil tests. Tests from the Cambridge Neuropsychological Test Automated Battery (CANTAB) (5), a battery of computerized cognitive tests that has been used in research on a variety of neurological disorders, including TBI (6–12), were included in the CENTER-TBI battery. The present study evaluates candidate cognitive endpoints, or composite scores, for the CENTER-TBI neuropsychological battery using data from the Trondheim MTBI follow-up study. In this study, as well as in the CENTER-TBI study, patients with mild traumatic brain injury (MTBI) were assessed 2 weeks after the injury. The extent of cognitive deficits 2 weeks after MTBI is uncertain, and empirical studies report effect sizes ranging from very small to medium (13–16).

A cognitive composite score combines several test scores into a single score (17). If an injury to the brain is associated with a deficit in one specific cognitive domain, it could be argued that this deficit will be washed out in a composite that includes measures of several domains, such as the overall mean score of a battery of tests. However, there is substantial variability between studies regarding which cognitive domains may be most affected after MTBI (18), suggesting heterogeneity in deficits between patients (e.g., some patients have attentional deficits and others have memory problems). Under these circumstances, a cognitive composite that considers each person's individual profile of low test scores might be well-suited for identifying deficits not only for the person, but also at the group level. A well-validated cognition composite score could serve as a primary or secondary endpoint in rehabilitation clinical trials (i.e., a summary variable that is used as a primary outcome to gauge the efficacy of an intervention), or as a variable of interest in a broad range of diagnostic or prognostic TBI studies involving neuroimaging and serum biomarkers. An endpoint that has strong psychometric properties would be sensitive to detecting small changes in cognitive functioning, which could be especially helpful in the TBI field given the variability of cognitive domains that could be affected by the injury (18). However, there is no well-validated and widely accepted cognition endpoint at present, neither in the TBI field in general (17), nor in the CENTER-TBI study. Seven candidate cognition summary scores have been developed in prior studies and applied to the Automated Neuropsychological Assessment Metrics (Version 4) Traumatic Brain Injury Military (ANAM4 TBI-MIL) (19) and the Delis-Kaplan Executive Function System (D-KEFS) (20) as part of a program of research designed to validate a cognition endpoint for TBI clinical trials (17). These previous studies evaluated a set of neuropsychological tests that are from the same publisher and are co-normed. However, in both research and clinical practice, neuropsychologists commonly administer a variety of tests that are standardized on different normative samples, and the composites have not yet been evaluated in neuropsychological test batteries that are not co-normed. Given that the test battery in CENTER-TBI consists of tests from different publishers and the study is conducted in multiple nations, a single normative group is not possible to use. Evaluating cognitive composites in a battery using different normative reference groups is important. The purpose of this study is to compare and contrast the seven cognition composite scores using the CENTER-TBI test battery in adults with MTBI, orthopedic injuries, and in healthy community controls. More specifically, we will (1) evaluate if some composites reveal greater group differences than others, and (2) assess the degree of intercorrelation between the composites to investigate whether certain endpoints could be considered redundant.

## Methods

### Participants

The participants in the present study were part of the Trondheim MTBI Follow-Up Study (total *N* = 378) (21). Patients with MTBI were recruited from April 2014 to December 2015. In the present study, adult patients were included if they were between ages 18 and 59 years and sustained a MTBI per the criteria described by the WHO Collaborating Center Task Force on MTBI: (a) mechanical energy to the head from external physical forces; (b) Glasgow Coma Scale (GCS) score of 13–15 at presentation to the emergency department; and (c) either witnessed loss of consciousness (LOC) <30 min, confusion, or post-traumatic amnesia (PTA) <24 h, or intracranial traumatic lesion not requiring surgery (22). Exclusion criteria were: (a) non-fluency in the Norwegian language; (b) pre-existing severe neurological (e.g., stroke, multiple sclerosis), psychiatric, somatic, or substance use disorders, determined to be severe enough to likely interfere with follow-up; (d) a prior history of a complicated mild, moderate, or severe TBI; (e) other concurrent major trauma (e.g., multiple fractures or internal bleeding) or brain injuries more severe than a MTBI.

Recruitment took place at a level 1 trauma center in Trondheim, Norway, and at the municipal emergency clinic, an outpatient clinic run by general practitioners. Patients were identified by daily screening of all referrals to head CT and patient lists at the municipal emergency clinic. Patients with a likely or possible MTBI were approached in the hospital ward or in the emergency department by study personnel, or contacted by telephone if they had left the emergency departments. LOC was self-reported and was categorized as present only if it was witnessed. Duration of PTA was also based on self-report and defined as the time after injury for which the patient had no continuous memory. It was dichotomized to either <1 or 1–24 h. A structured interview was conducted to assess LOC, PTA, and pre-injury health problems. Intracranial traumatic findings were obtained from Magnetic Resonance Imaging (MRI), performed within 72 h. MRI was performed on a 3.0 Tesla Siemens Skyra system (Siemens Healthcare, Erlangen, Germany) with a 32-channel head coil. The MTBI was classified as uncomplicated if there were no intracranial traumatic lesions on MRI, as described in detail previously (23). All patients in the present study underwent MRI. In addition, CT was performed in the majority of the patients, but none of the patients had intracranial findings on CT that were not detected on MRI (23).

Two control groups were recruited. One group consisted of patients with orthopedic injuries, free from trauma affecting the head, neck, or the dominant upper extremity (i.e., trauma controls). The trauma controls were identified by screening patient lists from the emergency departments. The other group consisted of healthy community controls. The community controls were recruited among hospital and university staff, students, and acquaintances of staff, students, and patients. The exclusion criteria were the same for the control groups and the MTBI group, but in addition, the community control group could not receive treatment for a serious psychiatric condition, even if they might be able to comply with follow-up. With this exception, medication in itself (e.g., analgesics) was not an exclusion criteria, neither for the MTBI group, nor for the control groups. The study was approved by the regional committee for research ethics (REK 2013/754) and was conducted in accordance with the Helsinki declaration. All participants gave informed consent.

### Neuropsychological Assessment

Participants with MTBI and trauma controls underwent neuropsychological testing approximately 2 weeks after the injury (MTBI: M = 16.6 days, SD = 3.1 days; Trauma controls: M = 17.1 days, SD = 3.4 days). The tests were administered by research staff with at least a Bachelor's degree in clinical psychology or neuroscience who were supervised by a licensed clinical psychologist. The total test time was around 90 min. The testing involved a larger battery, but in line with the purpose of the present study, only the tests included in the CENTER-TBI neuropsychological battery were analyzed in the current study. It should be noted that Norwegian norms do not exist for the CENTER-TBI battery and it is specified below which norms were used for each test. The Vocabulary subtest from the Norwegian version of the Wechsler Abbreviated Scale of Intelligence (WASI) was used to estimate premorbid intellectual functioning and raw scores were converted to age-referenced T scores using the normative data in the manual (24, 25). The Vocabulary subtest is commonly used for this purpose in TBI research because test performance is relatively unaffected by cognitive impairment following TBI (26, 27).

#### Paper-and-Pencil Tests

The traditional paper-and-pencil tests included in the CENTER-TBI battery are the Trail Making Test (TMT) Parts A and B and the Rey Auditory Verbal Test (RAVLT). On the TMT Part A (28), the task is to connect the numbers 1–25 with a line as fast as possible. On the TMT Part B, the participant is asked to draw a line alternating between numbers and letters as fast as possible. The outcome measure is time-to-completion and norms from Mitrushina et al. were used to calculate age-referenced T scores (29). On the RAVLT (28), the administrator reads a list of 15 words, and the participant is asked to recall as many words as possible. The test includes five trials. Then, an interference list is read and participants are asked to recall the words from the interference list. Thereafter, they are asked to recall the words from the original list immediately after the interference list, and again after 20 min. The sum of words remembered across the five trials and the number of words recalled following a 20-min delay (i.e., delayed recall score) were the outcome measures included in composite score calculation. Norms from Schmidt (30) published in Strauss et al. (28) were used to calculated age-referenced T scores. No stand-alone performance validity test was administered. For exploratory purposes, we examined rates of unusually low RAVLT scores that might reflect poor effort. Boone et al. (31) examined the RAVLT in patients suspected of giving non-credible memory performance based on their results on stand-alone performance validity tests. They reported that the Recognition Trial was the most useful for identifying possible poor effort. Given that the Recognition Trial was not administered as part of the CENTER TBI battery, we selected two other cutoff scores from Boone et al. that reflect unusually low scores that might reflect poor effort: Trial 5 ≤ 6 and Trials 1–5 ≤ 28. In our samples, the percentages of subjects who scored ≤6 on Trial 5 were as follows: MTBI = 2.1% (*n* = 3), trauma controls = 0%, and community controls = 0%. The percentages who scored ≤28 on Trials 1–5 were as follows: MTBI = 2.1% (*n* = 3), trauma controls = 1.4% (*n* = 1), and community controls = 0%. Given that there were no incentives to deliberately under-perform and the rates of these low scores were so low and of uncertain meaning, we did not exclude any subjects on the basis of these scores.

#### CANTAB Tests

The CANTAB involves a tablet-based assessment. The tests included in the CENTER-TBI battery are Attention Switching Task (AST), Paired Associates Learning (PAL), Rapid Visual Processing (RVP), Spatial Working Memory (SWM), Reaction Time Index (RTI), and Stockings of Cambridge (SOC). Raw scores were converted to age-referenced T scores using the CANTAB software. No norms are available for the AST (5), and this test was therefore not included in the present study (i.e., five CANTAB tests were included). Each CANTAB test generates up to 53 outcome measures (e.g., SWM). For inclusion in the composite scores, one outcome measure for each of the five tests was chosen. The outcome measure chosen for each test was the variable with normative data closest to being a total achievement/summary score in the “Recommended Measures Report” (5). On PAL, several boxes that contain different patterns are shown. Each pattern is subsequently shown for 1 s and the participant is asked to identify which box contains that pattern. “Total errors adjusted” was chosen as the outcome measure, with more errors indicative of worse performance (“adjusted” means that the score is adjusted for the number of trials completed). On RVP, participants are presented with numbers appearing on the screen at the rate of 100 digits per minute. The task is to press a button each time one of three target sequences (three digits) is shown. “A prime,” the outcome measure chosen, is a measure of the ability to identify the target sequence (i.e., the relationship between the probability of identifying a target sequence and the probability of identifying a non-target sequence). A higher score is indicative of better performance. On SWM, the task is to search through boxes for a token. When the token is found, a new token is placed in a different box. A token is not hidden in the same box twice; and to avoid errors, participants must remember where previous tokens appeared. The outcome measure chosen, “between errors,” is defined as the number of times the subject revisits a box in which a token has previously been found. A lower score is indicative of better performance. On RTI, the participant responds as fast as possible when a yellow dot is presented in one of five white circles. Response time in milliseconds was chosen as the outcome measure, with faster responding indicative of better performance. On SOC, two displays with three balls presented inside stockings appear on the screen, and the aim is to move the balls in one display such that it is identical to the arrangement of balls in the other display. The number of problems solved with the minimum possible moves was chosen as the outcome measure, with a higher score indicative of better performance.

### Composite Scores

Seven different composite scores, previously described in detail (19, 20), were calculated for the present study. Each composite score was calculated for the traditional paper-and-pencil tests only, the CANTAB tests only, and all tests (i.e., a combined composite), leading to 21 composites in total. All raw scores were converted to age-adjusted T scores (M = 50, SD = 10, in the normative sample), with higher scores indicative of better performance, before the composites, described below, were calculated. To avoid a disproportionate impact by unusual results on the composite scores, no subject was given a T score below 10 or above 90 (e.g., if a participant's score was converted to a T score of 9, this was set to 10). Only participants who completed all the nine tests included in the composite scores were included in the present study.

- The Overall Test Battery Mean (OTBM) was calculated by averaging T scores for all tests (32, 33). Lower scores equal worse performance.
- The Global Deficit Score (GDS) (34, 35) was calculated by assigning the following weights to T scores from each test: ≥40 = 0, 39–35 = 1, 34–30 = 2, 29–25 = 3, 24–20 = 4, and ≤19 = 5. Each participant's mean weight was then calculated for the each of the batteries. Higher scores equal worse performance.
- The Neuropsychological Deficit Score-Weighted (NDS-W) is a new composite calculated in previous cognition endpoint research only (19, 20). It assigns the following weights to T scores: ≥50 = 0, 49–47 = 0.25, 46–44 = 0.5, 43–41 = 1, 40–37 = 1.5, 36–35 = 2, 34–31 = 3, 30–28 = 4, 27–24 = 5, 23–21 = 6, and ≤20 = 7. The mean weight was then calculated for each of the batteries. Higher scores equal worse performance. This new deficit score is similar to the GDS, but provides an increase in gradations to lower the floor effect of the GDS.
- The Low Score Composite (LSC) is a new composite calculated in previous cognition endpoint research only (19, 20). T scores of 50 or higher are assigned a weight of 50, and T scores below 50 are assigned a weight that equals the T score (i.e., a T score of 40 would equal a weight of 40). The mean weight was then calculated for each of the batteries. Lower scores equal worse performance. This new composite score provides an even greater increase in gradation than the NDS-W.
- The number of scores at or below the 5th percentile (#≤ 5th %tile) is calculated by assigning the value 1 to scores at or below the 5th percentile (T score 34) and a zero to scores above the 5th percentile. These values are then summed for each participant. Higher scores equal worse performance. This score has been used in research calculating multivariate base rates for a range of neuropsychological test batteries (29–36).
- The number of scores at or below the 16th percentile (#≤ 16th %tile) is calculated by assigning the value 1 to scores at or below the 16th percentile (T score 40) and a zero to scores above the 16th percentile. These values are then summed for each participant. Higher scores equal worse performance. This score has also been calculated in previous multivariate base rate research (29–36).
- The number of scores below the 50th percentile (#< 50th %tile) is a new composite score, inspired by research on multivariate base rates, and previously calculated in cognition endpoint research only (19, 20). It is calculated by assigning the value 1 to scores below the 50th percentile (T score 49) and a zero to scores at or above the 50th percentile. These values are then summed for each participant. Higher scores equal worse performance.

### Statistical Analyses

Mann-Whitney *U*-tests and Kruskal-Wallis Rank Sum tests were used to examine differences in demographic variables, individual test scores, and in cognitive composite scores between the groups. These non-parametric methods were chosen because the majority of the composite scores showed non-normal distribution. The groups compared were: (1) participants with MTBI, trauma controls, and community controls; (2) participants with and without intracranial findings (i.e., complicated vs. uncomplicated MTBI); (3) participants with witnessed LOC and patients without LOC; (4) participants with long PTA (1–24 h) and participants with short PTA (<1 h). Cliff's delta was used to determine effect sizes between the groups on the composite scores. Cliff's delta is a measure of overlap between two distributions and is a suitable effect size measure for non-normal distributions (36). A Cliff's delta of 0 indicates complete overlap between the distributions, while a Cliff's delta of 1 or −1 indicate no overlap. As a guideline, a Cliff's delta of 0.11 is considered a small effect size, 0.28 a moderate effect size, and 0.43 a large effect size (37). Cohen's *d*s are also reported, but these should be interpreted with caution because of the non-normal distribution characterizing most of the composite scores. A Cohen's *d* of 0.20 is considered small, 0.50 moderate, and 0.80 large (38). The effect sizes were coded such that worse cognitive outcome in the presumed most affected group would result in a positive effect size. Notably, no corrections for multiple comparisons were applied because this study was designed to comprehensively explore a large number of candidate composite scores derived from the CENTER-TBI battery, with an emphasis on effect size interpretation as opposed to significance testing. Spearman's *rho* was used to examine the intercorrelations between the composite scores. All analyses were performed in IBM SPSS Statistics v. 25.

## Results

### Participant Characteristics

In total, 166 adults (≥18 years old) with MTBI were scheduled for the neuropsychological assessment 2 weeks following injury, as were 75 trauma controls and 74 community controls. Included in the present study are the 140 (84.3% of the scheduled patients) participants with MTBI, the 72 trauma controls (96.0%), and the 70 community controls (94.6%) who completed all nine tests constituting the composite scores. Demographic and clinical characteristics of the three groups are reported in Table 1. There were no statistically significant differences in age, sex, education, or estimated intelligence between the groups.

**Table 1:** Demographic and clinical characteristics of patients with MTBI, trauma controls, and community controls.

	**MTBI participants (*n* = 140)**	**Trauma controls (*n* = 72)**	**Community controls (*n* = 70)**	***p***
Age, mean (SD)	32.7 (12.2)	32.9 (12.8)	34.3 (12.6)	
Age, median (IQR)	28.7 (22.3–42.3)	28.8 (22.6–50.0)	29.6 (24.2–44.9)	0.463
Men, *n* (%)	96 (68.6)	46 (63.9)	44 (62.9)	0.650
Years of education, mean (SD)	14.2 (2.4)	14.6 (2.6)	14.5 (2.3)	
Years of education, median (IQR)	13 (12–16)	14 (12–16)	15 (13–16)	0.515
Estimated intelligence, T score, mean (SD)^a^	51.0 (9.1)	53.8 (6.7)	51.1 (8.5)	
Estimated intelligence, T score, median (IQR)^a^	52.0 (44.8–59.0)	53.0 (49.0–60.0)	50.0 (46.0–57.0)	0.088
Cause of injury, *n* (%)				
Fall	52 (37.1)	22 (30.6)	–	
Bicycle	27 (19.3)	7 (9.7)	–	
Violence	19 (13.6)	1 (1.4)	–	
Sport	16 (11.4)	28 (38.9)	–	
Motor vehicle accident	13 (9.3)	2 (2.8)	–	
Struck object	12 (8.6)	6 (8.3)	–	
Other	0 (0)	6 (8.3)	–	
Unknown	1 (0.7)	0 (0)	–	
GCS score, *n* (%)				
15	109 (77.9)	–	–	
14	19 (13.6)	–	–	
13	2 (1.4)	–	–	
Missing	10 (7.1)	–	–	
LOC, *n* (%)				
Yes, witnessed	68 (48.6)	–	–	
No	26 (18.6)	–	–	
Unknown/not witnessed	46 (32.9)	–	–	
PTA >1 h, *n* (%)	42 (30.0)	–	–	
Complicated MTBI, *n* (%)	17 (12.1)	–	–	
Level of care, *n* (%)				
Discharged home from ED	100 (71.4)	63 (87.5)	–	
Observation <24 h	23 (16.4)	–	–	
Neurosurgical admission	11 (7.9)	–	–	
Orthopedic/other admission	6 (4.3)	9 (12.5)	–	

*ED, Emergency Department; GCS, Glasgow Coma Scale; LOC, Loss of Consciousness; MTBI, Mild Traumatic Brain Injury; MVA, Motor Vehicle Accident; PTA, Posttraumatic Amnesia. Continuous variables were examined with Kruskal-Wallis Rank Sum tests and categorical variables with Chi-Square Test*.

a *Based on the Vocabulary subtest from Norwegian Version of the Wechsler Abbreviated Scale of Intelligence*.

### Characteristics of the Composite Scores and Group Comparisons

Scores from the individual tests that constitute the composite scores are reported in Table 2. There were no significant differences between the MTBI group, the trauma control group, and the community control group on any of the individual tests. Descriptive statistics and distributional characteristics for the composite scores in the MTBI group, the trauma control group, and the community control group are shown in Tables 3, 4, and the descriptive statistics for the composites in the severity subgroups (i.e., complicated MTBI, LOC, long PTA) are shown in Tables 5, 6. The OTBM and the #< 50th %tile composites had distributional characteristics (i.e., skewness and kurtosis) approaching a normal distribution, even if some deviations were seen (i.e., the CANTAB OTBM composite score had a skewness of −1.06 in the MTBI group). The other composites were, in general, highly skewed and zero-inflated (except the LCS, where 50 was the most common score). Group comparison (*p*-values and effect sizes) of the 21 composite scores are shown in Table 7. There were no significant differences between the MTBI group, the trauma control group, and the community control group on the composite scores and effect sizes were negligible (Cliff's delta <0.11) to small (Cliff's delta <0.28) for all composites. To investigate whether only including participants who completed all nine tests biased the results, we also calculated the OTBM (combined battery) for all participants who completed at least seven tests (i.e., the missing test score/s were replaced by the mean of that participant‘s available tests scores). The difference between this imputed OTBM and the one presented in the paper was negligible [for the MTBI group: 49.38 ± 6.59 (compared to the OTBM presented in Table 4: 49.64 ± 6.50); for the trauma control group 50.91 ± 5.80 (compared to the OTBM presented in Table 4: 51.08 ± 5.52); and for the community control group 50.82 ± 5.06 (compared to the OTBM presented in Table 4: 51.21 ± 4.93)].

**Table 2:** Descriptive statistics of the individual test scores.

	**Mild traumatic brain injury**	**Trauma controls**	**Community controls**	***p***
**TMT Part A**				
Raw, M, Md	26.86, 25.00	24.96, 25.00	24.89, 23.00	
Raw, SD	10.22	7.77	8.65	
Raw, interquartile range	20.00–31.00	18.25–29.00	19.00–30.00	
T score, M, Md	49.24, 51.32	51.45, 52.66	52.07, 54.22	0.173
T score, SD	11.17	8.92	8.87	
T score, interquartile range	43.51–56.92	45.36–57.82	47.91–58.98	
**TMT Part B**				
Raw, M, Md	64.90, 58.50	63.71, 56.50	61.14, 54.50	
Raw, SD	28.28	27.88	24.00	
Raw, interquartile range	45.25–74.75	47.25–71.75	42.00–75.75	
T score, M, Md	47.81, 50.48	49.12, 52.30	50.15, 52.32	0.543
T score, SD	12.40	9.54	9.65	
T score, interquartile range	43.11–56.66	44.49–55.40	44.74–57.91	
**RAVLT Trial 1–5**				
Raw, M, Md	50.37, 52.00	52.56, 54.00	52.14, 52.00	
Raw, SD	9.03	9.59	9.32	
Raw, interquartile range	44.00–56.00	46.25–60.00	44.50–60.00	
T score, M, Md	45.43, 47.56	48.58, 49.46	48.19, 47.61	0.185
T score, SD	11.69	11.57	11.60	
T score, interquartile range	38.22–53.15	41.44–56.35	40.58–58.63	
**RAVLT Delayed recall**				
Raw, M, Md	10.63, 11.00	11.46, 11.00	10.99, 12.00	
Raw, SD	2.92	2.68	2.91	
Raw, interquartile range	9.00–13.00	10.00–14.00	9.00–13.00	
T score, M, Md	48.88, 49.64	51.95, 52.86	50.42, 52.80	0.123
T score, SD	10.73	9.69	10.78	
T score, interquartile range	41.27–56.80	45.71–60.69	43.67–56.80	
**CANTAB—Paired associates learning**				
Raw, M, Md	8.61, 6.50	8.15, 5.00	9.03, 6.00	
Raw, SD	8.51	7.89	9.46	
Raw, interquartile range	3.00–11.00	3.00–12.00	2.00–12.00	
T score, M, Md	50.80, 52.53	51.47, 53.56	51.80, 53.87	0.553
T score, SD	7.32	5.95	6.55	
T score, interquartile range	47.44–55.29	47.81–55.21	48.75–56.58	
**CANTAB—Rapid visual processing**				
Raw, M, Md	0.91, 0.91	0.92, 0.92	0.91, 0.92	
Raw, SD	0.05	0.04	0.05	
Raw, interquartile range	0.88–0.94	0.90–0.95	0.88–0.94	
T score, M, Md	46.73, 47.56	49.85, 50.57	47.92, 50.88	0.250
T score, SD	12.10	10.16	10.44	
T score, interquartile range	38.55–55.92	43.81–55.71	42.04–54.90	
**CANTAB—Spatial working memory**				
Raw, M, Md	13.67, 10.50	15.74, 11.50	16.44, 10.50	
Raw, SD	13.44	15.64	16.97	
Raw, interquartile range	2.00–22.00	2.00–26.75	2.00–29.75	
T score, M, Md	53.07, 55.28	51.72, 53.82	52.91, 54.12	0.665
T score, SD	8.72	9.48	8.02	
T score, interquartile range	49.07–59.72	43.95–59.72	48.99–59.72	
**CANTAB—Reaction time index**				
Raw, M, Md	336.9, 331.9	328.4, 327.3	321.8, 316.9	
Raw, SD	57.2	40.6	43.3	
Raw, interquartile range	300.5–354.5	302.3–349.8	294.9–346.7	
T score, M, Md	52.37, 53.46	53.77, 53.52	55.41, 56.47	0.086
T score, SD	9.28	8.07	8.23	
T score, interquartile range	48.32–59.04	49.05–58.77	50.01–61.01	
**CANTAB—Stockings of Cambridge**				
Raw, M, Md	9.62, 10.00	9.49, 10.00	9.43, 10.00	
Raw, SD	1.79	1.99	1.98	
Raw, interquartile range	8.00–11.00	8.00–11.00	8.00–11.00	
T score, M, Md	52.44, 53.66	51.78, 52.87	51.98, 54.23	0.845
T score, SD	9.65	10.20	9.79	
T score, interquartile range	46.89–59.38	43.92–59.41	46.38–59.38	

*CANTAB, The Cambridge Neuropsychological Test Automated Battery; RAVLT, Rey Auditory Verbal Learning Test; Raw, Raw score; TMT, Trail Making Test. Group differences were examined with Kruskal-Wallis Rank Sum tests*.

**Table 3:** Descriptive statistics of the seven composite scores based on the traditional paper and pencil tests (4 individual test scores) and the CANTAB tests (5 individual test scores).

	**Paper-and-pencil composites**			**CANTAB composites**		
	**MTBI**	**Trauma controls**	**Community controls**	**MTBI**	**Trauma controls**	**Community controls**
**OTBM**						
M, Md	47.84, 49.03	50.28, 51.42	50.21, 50.80	51.08, 51.62	51.72, 51.66	52.00, 52.52
SD	8.87	7.06	7.37	6.18	5.87	5.34
Interquartile range	42.43–54.42	45.72–55.40	43.91–55.35	48.01–55.93	48.18–56.04	48.80–55.05
Range (Min–Max)	21.32–63.13	31.93–64.51	34.14–64.63	25.76–61.47	38.55–62.29	39.27–61.83
Skewness, kurtosis	−0.77, 0.27	−0.45, −0.32	−0.16, −0.69	−1.06, 1.88	−0.26, −0.56	−0.31, −0.36
**GDS**						
Percent scoring zero	57.9	58.3	57.1	60.7	68.1	62.9
M, Md	0.50, 0.00	0.28, 0.00	0.33, 0.00	0.26, 0.00	0.18, 0.00	0.20, 0.00
SD	0.84	0.48	0.50	0.50	0.34	0.34
Interquartile range	0.00–0.75	0.00–0.50	0.00–0.50	0.00–0.40	0.00–0.35	0.00–0.20
Range (Min–Max)	0.00–3.50	0.00–2.00	0.00–2.00	0.00–3.20	0.00–1.60	0.00–1.20
Skewness, kurtosis	1.97, 3.37	2.05, 3.60	1.54, 1.57	2.98, 11.03	2.19, 4.77	1.69, 1.74
**NDS-W**						
Percent scoring zero	17.9	19.4	22.9	12.1	20.8	22.9
M, Md	0.94, 0.41	0.60, 0.25	0.68, 0.35	0.56, 0.25	0.45, 0.30	0.44, 0.25
SD	1.22	0.77	0.78	0.75	0.55	0.54
Interquartile range	0.06–0.1.25	0.06–0.88	0.06–1.13	0.10–0.70	0.05–0.64	0.05–0.61
Range (Min–Max)	0.00–5.25	0.00–3.13	0.00–2.88	0.00–4.45	0.00–2.40	0.00–2.05
Skewness, kurtosis	1.72, 2.50	1.69, 2.30	1.17, 0.42	2.40, 7.00	2.19, 4.77	1.44, 1.11
**LSC**						
Percent scoring 50	17.9	19.4	22.9	12.1	20.8	22.9
M, Md	44.49, 46.91	46.20, 47.75	45.88, 47.35	46.70, 47.94	47.20, 48.02	47.30, 48.47
SD	6.49	4.46	4.32	4.02	3.12	3.11
Interquartile range	41.71–49.50	44.16–49.84	43.17–49.82	45.47–49.40	46.26–49.74	46.08–49.96
Range (Min–Max)	21.32–50.00	31.93–50.00	34.14–50.00	25.76–50.00	37.25–50.00	38.51–50.00
Skewness, kurtosis	−1.53, 2.03	−1.34, 1.10	−0.91, −0.23	−2.25, 6.73	−1.33, 1.04	−1.20, 0.47
**#≤ 5th %tile**						
Percent scoring zero	68.6	79.2	72.9	74.3	79.2	78.6
M, Md	0.54, 0.00	0.29, 0.00	0.37, 0.00	0.35, 0.00	0.26, 0.00	0.26, 0.00
SD	0.91	0.62	0.66	0.69	0.56	0.53
Interquartile range	0.00–1.00	0.00–0.00	0.00–1.00	0.00–1.00	0.00–0.00	0.00–0.00
Range (Min–Max)	0.00–4.00	0.00–2.00	0.00–2.00	0.00–4.00	0.00–2.00	0.00–2.00
Skewness, kurtosis	1.64, 1.86	1.97, 2.62	1.57, 1.12	2.37, 6.72	2.04, 3.24	1.99, 3.20
**#≤ 16th %tile**						
Percent scoring zero	50.7	55.6	51.4	57.9	61.1	61.4
M, Md	0.97, 0.00	0.71, 0.00	0.86, 0.00	0.68, 0.00	0.65, 0.00	0.60, 0.00
SD	1.22	0.96	1.03	0.96	0.99	0.87
Interquartile range	0.00–2.00	0.00–1.00	0.00–2.00	0.00–1.00	0.00–1.00	0.00–1.00
Range (Min–Max)	0.00–4.00	0.00–3.00	0.00–3.00	0.00–4.00	0.00–4.00	0.00–3.00
Skewness, kurtosis	1.08, 0.12	1.22, 0.45	0.79, −0.71	1.47, 1.83	1.55, 1.64	1.29, 0.65
**#< 50th %tile**						
Percent scoring zero	17.9	19.4	22.9	12.1	20.8	22.9
M, Md	2.04, 2.00	1.78, 2.00	1.73, 2.00	1.97, 2.00	1.90, 2.00	1.67. 1.50
SD	1.43	1.30	1.35	1.27	1.40	1.37
Interquartile range	1.00–3.00	1.00–3.00	1.00–3.00	1.00–3.00	1.00–3.00	1.00–2.25
Range (Min–Max)	0.00–4.00	0.00–4.00	0.00–4.00	0.00–5.00	0.00–5.00	0.00–5.00
Skewness, kurtosis	0.04, −1.34	0.27, −0.93	0.29, −0.71	0.31, −0.56	0.18, −0.90	0.59, −0.42

*#, Number of scores; CANTAB, Cambridge Neuropsychological Test Automated Battery; GDS, Global Deficit Score; LSC, Low Score Composite; MTBI, Mild Traumatic Brain Injury; NDS-W, Neuropsychological Deficit Score-Weighted; OTBM, Overall Test Battery Mean. A normal distribution has a skewness and kurtosis of 0*.

**Table 4:** Descriptive statistics of the combined composite scores (based on all 9 individual test scores).

	**MTBI**	**Trauma controls**	**Community controls**
**OTBM**			
M, Md	49.64, 50.67	51.08, 51.92	51.21, 51.47
SD	6.50	5.52	4.93
Interquartile range	45.95–54.33	47.17–54.79	47.60–54.82
Range (Min–Max)	30.17–60.01	35.61–62.86	39.06–62.98
Skewness, kurtosis	−0.78, 0.41	−0.56, 0.07	−0.27, −0.42
**GDS**			
Percent scoring zero	42.1	43.1	38.6
M, Md	0.37, 0.11	0.23, 0.11	0.26, 0.17
SD	0.57	0.33	0.30
Interquartile range	0.00–0.44	0.00–0.22	0.00–0.44
Range (Min–Max)	0.00–2.67	0.00–1.67	0.00–1.11
Skewness, kurtosis	2.07, 4.09	2.16, 5.19	1.07, 0.13
**NDS-W**			
Percent scoring zero	2.9	8.3	8.6
M, Md	0.73, 0.42	0.52, 0.32	0.54, 0.40
SD	0.84	0.56	0.50
Interquartile range	0.14–0.97	0.15–0.69	0.13–0.83
Range (Min–Max)	0.00–3.69	0.00–2.72	0.00–1.92
Skewness, kurtosis	1.74, 2.67	1.76, 3.33	0.90, −0.07
**LSC**			
Percent scoring 50	2.9	8.3	8.6
M, Md	45.72, 47.14	46.75, 47.88	46.67, 47.15
SD	4.46	3.22	2.87
Interquartile range	43.94–49.01	45.19–48.94	44.97–49.11
Range (Min–Max)	29.96–50.00	34.88–50.00	38.64–50.00
Skewness, kurtosis	−1.58, 2.32	−1.46, 1.99	−0.76, −0.24
**#≤ 5th %tile**			
Percent scoring zero	57.9	65.3	58.6
M, Md	0.89, 0.00	0.56, 0.00	0.62, 0.00
SD	1.37	0.95	0.85
Interquartile range	0.00–1.00	0.00–1.00	0.00–1.00
Range (Min–Max)	0.00–7.00	0.00–4.00	0.00–3.00
Skewness, kurtosis	1.83, 3.36	2.03, 4.08	1.09, 0.10
**#≤ 16th %tile**			
Percent scoring zero	37.1	38.9	34.3
M, Md	1.65, 1.00	1.36, 1.00	1.46, 1.00
SD	1.81	1.59	1.42
Interquartile range	0.00–3.00	0.00–2.00	0.00–2.00
Range (Min–Max)	0.00–7.00	0.00–6.00	0.00–5.00
Skewness, kurtosis	1.04, 0.22	1.23, 0.64	0.76, −0.17
**#< 50th %tile**			
Percent scoring zero	2.9	8.3	8.6
M, Md	4.01. 4.00	3.68, 3.00	3.40, 3.00
SD	2.29	2.25	2.23
Interquartile range	2.00–6.00	2.00–5.00	2.00–5.00
Range (Min–Max)	0.00–9.00	0.00–8.00	0.00–8.00
Skewness, kurtosis	0.18, −0.94	0.40, −0.57	0.31, −0.90

*#, Number of scores; GDS, Global Deficit Score; LSC, Low Score Composite; MTBI, Mild Traumatic Brain Injury; NDS-W, Neuropsychological Deficit Score-Weighted; OTBM, Overall Test Battery Mean. A normal distribution has a skewness and kurtosis of 0*.

**Table 5:** Descriptive statistics of the traditional paper-and-pencil composite scores and the CANTAB composite scores in the severity subgroups.

	**Complicated *n* = 17**	**Uncomplicated *n* = 123**	**LOC *n* = 68**	**No LOC *n* = 26**	**Long PTA *n* = 42**	**Short PTA *n* = 98**
**PAPER-AND-PENCIL**						
**OTBM**						
M, Md	45.96, 48.21	48.10, 49.10	48.39, 48.65	48.39, 50.02	44.92, 46.36	49.09, 50.50
SD	11.76	8.42	7.70	10.02	9.54	8.30
Interquartile range	39.09–55.21	42.63–54.49	44.65–54.21	41.93–56.58	38.43–52.53	44.91–55.37
**GDS**						
M, Md	0.82, 0.25	0.46, 0.00	0.42, 0.00	0.55, 0.00	0.73, 0.25	0.41, 0.00
SD	1.19	0.78	0.73	0.97	0.99	0.76
Interquartile range	0.00–1.50	0.00–0.50	0.00–0.50	0.00–0.81	0.00–1.50	0.00–0.50
**NDS-W**						
M, Md	1.30, 0.50	0.89, 0.38	0.82, 0.44	0.99, 0.28	1.29, 0.75	0.79, 0.25
SD	1.64	1.15	1.06	1.41	0.99	1.12
Interquartile range	0.03–2.19	0.06–1.19	0.13–1.17	0.05–1.34	0.00–1.50	0.06–1.19
**LSC**						
M, Md	42.59, 46.45	44.76, 47.26	45.09, 46.86	44.44, 47.79	42.46, 44.89	45.37, 47.97
SD	9.08	6.05	5.63	7.19	7.40	5.89
Interquartile range	38.70–49.74	42.02–49.51	42.69–49.17	41.91–49.75	37.85–48.71	43.10–49.68
**#≤ 5th %tile**						
M, Md	0.82, 0.00	0.50, 0.00	0.46, 0.00	0.58, 0.00	0.76, 0.00	0.44, 0.00
SD	1.13	0.87	0.85	1.06	1.03	0.84
Interquartile range	0.00–2.00	0.00–1.00	0.00–1.00	0.00–1.00	0.00–2.00	0.00–1.00
**#≤ 16th %tile**						
M, Md	1.06, 1.00	0.96, 0.00	0.87, 0.00	0.96, 0.00	1.38, 1.00	0.80, 0.00
SD	1.25	1.22	1.12	1.31	1.32	1.13
Interquartile range	0.00–2.00	0.00–2.00	0.00–1.75	0.00–2.00	0.00–2.25	0.00–1.00
**#< 50th %tile**						
M, Md	2.00, 2.00	2.04, 2.00	1.97, 2.00	1.85, 1.50	2.55, 3.00	1.82, 1.50
SD	1.50	1.43	1.34	1.52	1.42	1.39
Interquartile range	0.50–3.00	1.00–3.00	1.00–3.00	0.75–3.25	2.00.4.00	1.00–3.00
**CANTAB**						
**OTBM**						
M, Md	51.40, 52.44	51.04, 51.59	51.05, 52.03	50.71, 52.08	48.86, 49.44	52.03, 52.85
SD	6.95	6.09	6.44	5.65	5.91	6.07
Interquartile range	49.18–57.19	47.76–55.08	48.13–56.29	47.76–54.92	45.22–52.81	49.91, 56.37
**GDS**						
M, Md	0.31, 0.00	0.26, 0.00	0.26, 0.00	0.28, 0.00	0.38, 0.20	0.21, 0.00
SD	0.62	0.49	0.53	0.49	0.52	0.49
Interquartile range	0.00–0.20	0.00–0.40	0.00–0.40	0.00–0.45	0.00–0.60	0.00–0.20
**NDS-W**						
M, Md	0.55, 0.20	0.56, 0.30	0.57, 0.30	0.56, 0.23	0.77, 0.50	0.47, 0.20
SD	0.89	0.74	0.79	0.69	0.79	0.72
Interquartile range	0.03–0.48	0.10–0.85	0.05–0.81	0.10–0.89	0.10–1.21	0.09–0.50
**LSC**						
M, Md	46.89, 48.44	46.67, 47.86	46.57, 47.71	46.70, 48.28	45.49, 46.29	47.18, 48.57
SD	4.52	3.97	4.35	3.82	4.07	3.93
Interquartile range	46.46–49.97	45.29–49.38	45.33–49.49	44.60–49.12	42.84–49.19	46.71–49.47
**#≤ 5th %tile**						
M, Md	0.29, 0.00	0.36, 0.00	0.35, 0.00	0.42, 0.00	0.55, 0.00	0.27, 0.00
SD	0.69	0.69	0.73	0.64	0.74	0.65
Interquartile range	0.00–0.00	0.00–1.00	0.00–1.00	0.00–1.00	0.00–1.00	0.00–0.00
**#≤ 16th %tile**						
M, Md	0.71, 0.00	0.67, 0.00	0.69, 0.00	0.69, 0.00	1.00, 1.00	0.54, 0.00
SD	1.10	0.95	0.95	1.09	1.08	0.88
Interquartile range	0.00–1.00	0.00–1.00	0.00–1.00	0.00–1.00	0.00–1.00	0.00–1.00
**#< 50th %tile**						
M, Md	1.65, 1.00	2.02, 2.00	1.99, 2.00	2.08, 2.00	2.48, 3.00	1.76, 2.00
SD	1.37	1.26	1.26	1.16	1.31	1.20
Interquartile range	0.50–3.00	1.00–3.00	1.00–3.00	1.00–3.00	1.75–3.00	1.00–3.00

*#, Number of scores; CANTAB, Cambridge Neuropsychological Test Automated Battery; Complicated, intracranial findings on MRI; GDS, Global Deficit Score; LOC, Loss of Consciousness; LSC, Low Score Composite; MTBI, Mild Traumatic Brain Injury; NDS-W, Neuropsychological Deficit Score-Weighted; OTBM, Overall Test Battery Mean; PTA, Posttraumatic Amnesia*.

**Table 6:** Descriptive statistics of the combined composite scores in the severity subgroups.

	**Complicated *n* = 17**	**Uncomplicated *n* = 123**	**LOC *n* = 68**	**No LOC *n* = 26**	**Long PTA *n* = 42**	**Short PTA *n* = 98**
**OTBM**						
M, Md	48.98, 50.34	49.73, 50.79	49.87, 51.52	49.68, 51.49	47.11, 47.82	50.73, 51.99
SD	8.05	6.29	6.24	6.79	6.44	6.24
Interquartile range	45.21–55.34	45.93–54.37	46.63–54.10	45.57–55.13	42.25–52.48	47.54–55.13
**GDS**						
M, Md	0.54, 0.22	0.35, 0.11	0.34, 0.11	0.40, 0.06	0.53, 0.22	0.30, 0.11
SD	0.75	0.54	0.56	0.59	0.61	0.53
Interquartile range	0.00–0.78	0.00–0.33	0.00–0.33	0.00–0.61	0.00–1.00	0.00–0.33
**NDS-W**						
M, Md	0.88, 0.53	0.70, 0.42	0.68, 0.39	0.75, 40	1.00, 0.78	0.61, 0.26
SD	1.07	0.80	0.82	0.87	0.88	0.79
Interquartile range	0.10–1.19	0.14–0.92	0.17–0.92	0.13–1.14	0.26–1.71	0.11–0.79
**LSC**						
M, Md	44.98, 46.81	45.82, 47.26	45.91, 47.60	45.70, 47.59	44.20, 45.33	46.37, 48.06
SD	5.67	4.28	4.41	4.60	4.63	4.24
Interquartile range	43.08–49.21	44.05–49.01	44.56–48.92	43.63–49.00	40.49–48.06	44.96–49.20
**#≤ 5th %tile**						
M, Md	1.12, 0.00	0.85, 0.00	0.81, 0.00	1.00, 0.00	1.31, 1.00	0.70, 0.00
SD	1.58	1.34	1.37	1.44	1.46	1.29
Interquartile range	0.00–2.00	0.00–1.00	0.00–1.00	0.00–1.25	0.00–2.25	0.00–1.00
**#≤ 16th %tile**						
M, Md	1.76, 1.00	1.63, 1.00	1.56, 1.00	1.65, 1.00	2.38, 2.00	1.34, 1.00
SD	1.99	1.79	1.81	2.02	1.94	1.67
Interquartile range	0.00–2.50	0.00–3.00	0.00–2.00	0.00–3.00	1.00–4.00	0.00–2.00
**#< 50th %tile**						
M, Md	3.65, 4.00	4.06, 4.00	3.96, 4.00	3.92, 3.00	5.02, 5.00	3.57, 3.00
SD	2.42	2.28	2.18	2.43	2.31	2.15
Interquartile range	1.50–5.50	2.00–6.00	2.25–6.00	2.00–6.00	3.00–7.00	2.00–5.00

*#, Number of scores; GDS, Global Deficit Score; LOC, Loss of Consciousness; LSC, Low Score Composite; MTBI, Mild Traumatic Brain Injury; NDS-W, Neuropsychological Deficit Score-Weighted; OTBM, Overall Test Battery Mean; PTA, Posttraumatic Amnesia*.

**Table 7:** Group comparisons, *p*-values and effect sizes [Cliff's delta (Cohen's *d*)].

	**MTBI/trauma control/community control**			**Complicated**		**LOC**		**PTA**	
	***p***	**Effect sizes: MTBI vs. trauma controls**	**Effect sizes: MTBI vs. community controls**	***p***	**Effect sizes**	***p***	**Effect sizes**	***p***	**Effect sizes**
**Paper-and-Pencil**									
OTBM	0.152	0.14 (0.30)	0.12 (0.28)	0.603	0.07 (0.24)	0.685	0.05 (0.00)	0.013	0.27 (0.48)
GDS	0.655	0.06 (0.31)	0.03 (0.23)	0.221	0.16 (0.43)	0.889	−0.02 (−0.16)	0.054	0.18 (0.39)
NDS-W	0.295	0.12. (0.32)	0.08 (0.24)	0.593	0.08 (0.34)	0.799	0.03 (−0.15)	0.020	0.25 (0.49)
LSC	0.316	0.12 (0.29)	0.08 (0.24)	0.625	0.07 (0.25)	0.786	0.04 (−0.11)	0.017	0.25 (0.46)
#≤ 5th %tile	0.186	0.12 (0.31)	0.06 (0.21)	0.243	0.14 (0.35)	0.671	−0.05 (−0.13)	0.071	0.16 (0.36)
#≤ 16th %tile	0.471	0.09 (0.23)	0.03 (0.10)	0.710	0.05 (0.08)	0.960	−0.01 (−0.08)	0.008	0.26 (0.49)
#< 50th %tile	0.261	0.10 (0.18)	0.12 (0.22)	0.889	−0.02 (−0.03)	0.660	0.06 (0.08)	0.006	0.29 (0.52)
**CANTAB**									
OTBM	0.733	0.03 (0.11)	0.07 (0.16)	0.681	−0.06 (−0.06)	0.806	−0.03 (−0.05)	0.001	0.37 (0.53)
GDS	0.582	0.08 (0.18)	0.03 (0.13)	0.780	−0.04 (0.10)	0.908	0.01 (−0.04)	0.009	0.24 (0.34)
NDS-W	0.468	0.06 (0.16)	0.10 (0.18)	0.314	−0.15 (−0.01)	0.728	−0.05 (0.01)	0.015	0.26 (0.41)
LSC	0.458	0.06 (0.13)	0.10 (0.16)	0.370	−0.13 (−0.05)	0.819	−0.03 (0.03)	0.013	0.27 (0.43)
#≤ 5th %tile	0.628	0.05 (0.14)	0.05 (0.14)	0.520	−0.07 (−0.10)	0.426	−0.08 (−0.10)	0.007	0.22 (0.41)
#≤ 16th %tile	0.862	0.03 (0.03)	0.04 (0.08)	0.946	−0.01 (0.05)	0.752	0.04 (0.00)	0.008	0.25 (0.49)
#< 50th %tile	0.239	0.03 (0.05)	0.14 (0.23)	0.265	−0.16 (−0.29)	0.903	−0.02 (−0.07)	0.002	0.31 (0.58)
**Combined**									
OTBM	0.259	0.11 (0.23)	0.11 (0.30)	0.757	0.04 (0.12)	0.866	0.02 (−0.03)	0.001	0.35 (0.57)
GDS	0.541	0.08 (0.29)	0.01 (0.23)	0.469	0.10 (0.34)	0.950	−0.01 (−0.11)	0.005	0.29 (0.42)
NDS-W	0.407	0.11 (0.28)	0.07 (0.26)	0.861	0.03 (0.22)	0.990	0.00 (−0.09)	0.003	0.32 (0.48)
LSC	0.426	0.10 (0.26)	0.07 (0.24)	0.916	0.02 (0.19)	0.886	0.02 (−0.05)	0.003	0.32 (0.50)
#≤ 5th %tile	0.355	0.10 (0.27)	0.04 (0.23)	0.537	0.08 (0.20)	0.518	−0.08 (−0.14)	0.007	0.26 (0.45)
#≤ 16th %tile	0.610	0.08 (0.17)	0.01 (0.11)	0.841	0.03 (0.07)	0.937	0.01 (−0.05)	0.001	0.33 (0.59)
#< 50th %tile	0.199	0.08 (0.14)	0.15 (0.27)	0.535	−0.09 (−0.18)	0.739	0.04 (0.02)	0.001	0.36 (0.66)

*#, Number of scores; CANTAB, Cambridge Neuropsychological Test Automated Battery; Effect size, Cliff's delta (Cohen's d); GDS, Global Deficit Score; LOC, Loss of Consciousness; LSC, Low Score Composite; MTBI, Mild Traumatic Brain Injury; NDS-W, Neuropsychological Deficit Score-Weighted; OTBM, Overall Test Battery Mean; PTA, Posttraumatic Amnesia. P-values are from Kruskal-Wallis Tests for the three-group comparisons and Mann-Whitney U tests for the two-group comparisons. Cliff's delta is presented first followed by Cohen's d in parentheses. A positive effect sized indicates lower performance in the group assumed to have the greatest deficits (i.e., MTBI, Complicated; LOC, Long PTA). A Cliff's delta of 0.11 is considered a small effect size, 0.28 a moderate effect size, and 0.43 a large effect size. A Cohen's d of 0.2 is considered small, 0.5 moderate, and 0.8 large. Cohen's ds should be interpreted with caution because of the non-normal distribution characterizing most of the composite scores*.

### Severity Subgroups

There were no significant differences in age (*p* = 0.745), sex (*p* = 0.560), education (*p* = 0.354), or estimated intelligence (*p* = 0.491) between participants with complicated MTBI and uncomplicated MTBI. None of composites revealed statistically significant differences between the groups and effect sizes (Cliff's delta) were negligible to small (Table 7). Similarly, there were no significant differences in age (*p* = 0.852), sex (*p* = 0.489), education (*p* = 0.542), or estimated intelligence (*p* = 0.802) between participants with and without LOC, and none of the composites revealed statistically significant differences between the groups (Table 7). Participants with long PTA had significantly lower estimated intelligence than participants with short PTA (long PTA: M = 48.1, SD = 9.3; short PTA: M = 52.2, SD = 8.8; *p* = 0.011), but there were no significant differences in age (*p* = 0.181), sex (*p* = 0.266), or education (*p* = 0.101). All composites except the paper-and-pencil GDS and #≤ 5th %tile differed significantly between participants with long and short PTA, with lower cognitive functioning in participants with long PTA (Table 7). Effect sizes ranged from small to moderate. On the combined composites, the largest effect sizes (Cliff's delta) were observed on the #< 50th %tile (0.36) and the OTBM (0.35) composites and the smallest on the #≤ 5th %tile (0.26) composite. Similar effect sizes (small to moderate) were seen on the paper-and-pencil composites and on the CANTAB composites.

### Intercorrelations of the Composite Scores

In general, the intercorrelations between the composite scores were high on analyses that stratified the data by group (i.e., MTBI; trauma control; community control) and test battery (i.e., paper-and-pencil, the CANTAB, and combined; Tables 8, 9). For example, in the MTBI group, the correlations between the composite scores on the combined battery ranged from 0.68 (between the #≤ 5th %tile and the #< 50th %tile) to −0.99 (between the NDS-W and the LSC). Correlations *between* the paper-and-pencil composites and the CANTAB composites were lower, ranging in the MTBI group from 0.55 (between the paper-and-pencil and CANTAB OTBM composites) to 0.33 (between the paper-and-pencil #≤ 5th %tile and the CANTAB #≤ 16th %tile composites). In the control groups, the correlations were somewhat lower between the paper-and-pencil composites and the CANTAB composites. For example, the correlation between the paper-and-pencil #≤ 5th %tile and the CANTAB #≤ 16th %tile composites was 0.26 in the trauma control group and 0.09 in the community control group. Correlations between estimated intelligence (the Vocabulary subtest) and the composites scores are also shown in Tables 8, 9, and most composites were significantly correlated with estimated intelligence.

**Table 8:** Spearman intercorrelation matrices for the composite scores in the MTBI group.

		**1**	**2**	**3**	**4**	**5**	**6**	**7**	**8**	**9**	**10**	**11**	**12**	**13**	**14**	**15**	**16**	**17**	**18**	**19**	**20**	**21**
**Paper-and-pencil**	1. OTBM	1																				
	2. GDS	−0.84^a^	1																			
	3. NDS-W	−0.96^a^	0.88^a^	1																		
	4. LSC	0.97^a^	−0.88^a^	−0.99^a^	1																	
	5. #≤ 5th %tile	−0.76^a^	0.91^a^	0.81^a^	−0.79^a^	1																
	6. #≤ 16th %tile	−0.89^a^	0.91^a^	0.91^a^	−0.91^a^	0.81^a^	1															
	7. #< 50th %tile	−0.90^a^	0.65^a^	0.86^a^	−0.88^a^	0.55^a^	0.76^a^	1														
**CANTAB**	8. OTBM	0.55^a^	−0.42^a^	−0.51^a^	0.52^a^	−0.39^a^	−0.44^a^	−0.47^a^	1													
	9. GDS	−0.45^a^	0.40^a^	0.44^a^	−0.44^a^	0.37^a^	0.41^a^	0.38^a^	−0.80^a^	1												
	10. NDS-W	−0.50^a^	0.40^a^	0.46^a^	−0.46^a^	0.37^a^	0.42^a^	0.42^a^	−0.93^a^	0.86^a^	1											
	11. LSC	0.49^a^	−0.39^a^	−0.45^a^	0.46^a^	−0.36^a^	−0.41^a^	−0.42^a^	0.92^a^	−0.86^a^	−0.99^a^	1										
	12. #≤ 5th %tile	−0.46^a^	0.43^a^	0.46^a^	−0.46^a^	0.40^a^	0.45^a^	0.39^a^	−0.72^a^	0.87^a^	0.76^a^	−0.75^a^	1									
	13. #≤ 16th %tile	−0.43^a^	0.36^a^	0.42^a^	−0.42^a^	0.33^a^	0.38^a^	0.36^a^	−0.81^a^	0.95^a^	0.87^a^	−0.87^a^	0.78^a^	1								
	14. #< 50th %tile	−0.50^a^	0.37^a^	0.46^a^	−0.46^a^	0.38^a^	0.39^a^	0.43^a^	−0.90^a^	0.67^a^	0.86^a^	−0.86^a^	0.60^a^	0.69^a^	1							
**Combined**	15. OTBM	0.90^a^	−0.73^a^	−0.85^a^	0.86^a^	−0.67^a^	−0.77^a^	−0.80^a^	0.85^a^	−0.68^a^	−0.77^a^	0.77^a^	−0.64^a^	−0.66^a^	−0.77^a^	1						
	16. GDS	−0.81^a^	0.87^a^	0.84^a^	−0.83^a^	0.80^a^	0.83^a^	0.65^a^	−0.67^a^	0.73^a^	0.68^a^	−0.67^a^	0.68^a^	0.70^a^	0.58^a^	−0.85^a^	1					
	17. NDS-W	−0.89^a^	0.81^a^	0.90^a^	−0.90^a^	0.75^a^	0.83^a^	0.78^a^	−0.77^a^	0.70^a^	0.76^a^	−0.75^a^	0.67^a^	0.68^a^	0.69^a^	−0.95^a^	0.93^a^	1				
	18. LSC	0.90^a^	−0.80^a^	−0.89^a^	0.90^a^	−0.73^a^	−0.83^a^	−0.79^a^	0.78^a^	−0.70^a^	−0.77^a^	0.77^a^	−0.65^a^	−0.69^a^	−0.71^a^	0.96^a^	−0.92^a^	−0.99^a^	1			
	19. #≤ 5th %tile	−0.76^a^	0.83^a^	0.80^a^	−0.78^a^	0.87^a^	0.79^a^	0.60^a^	−0.61^a^	0.65^a^	0.61^a^	−0.60^a^	0.74^a^	0.59^a^	0.55^a^	−0.79^a^	0.92^a^	0.87^a^	−0.84^a^	1		
	20. #≤ 16th %tile	−0.84^a^	0.80^a^	0.84^a^	−0.85^a^	0.71^a^	0.87^a^	0.72^a^	−0.71^a^	0.74^a^	0.72^a^	−0.71^a^	0.67^a^	0.75^a^	0.62^a^	−0.88^a^	0.94^a^	0.94^a^	−0.94^a^	0.84^a^	1	
	21. #< 50th %tile	−0.85^a^	0.62^a^	0.80^a^	−0.81^a^	0.56^a^	0.70^a^	0.87^a^	−0.79^a^	0.61^a^	0.74^a^	−0.74^a^	0.57^a^	0.61^a^	0.81^a^	−0.93^a^	0.74^a^	0.88^a^	−0.89^a^	0.68^a^	0.80^a^	1
	22. Vocabulary	0.38^a^	−0.24^a^	−0.30^a^	0.31^a^	−0.24^a^	−0.31^a^	−0.33^a^	0.47^a^	−0.29^a^	−0.43^a^	0.43^a^	−0.25^a^	−0.26^a^	−0.45^a^	0.48^a^	−0.32^a^	−0.40^a^	0.42^a^	−0.29^a^	−0.35^a^	−0.44^a^

#, Number of scores; GDS, Global Deficit Score; LSC, Low Score Composite; NDS-W, Neuropsychological Deficit Score-Weighted; OTBM, Overall Test Battery Mean;

a *p < 0.01*.

**Table 9:** Spearman intercorrelation matrices for the composite scores in the trauma control group and the community control group.

		**1**	**2**	**3**	**4**	**5**	**6**	**7**	**8**	**9**	**10**	**11**	**12**	**13**	**14**	**15**	**16**	**17**	**18**	**19**	**20**	**21**
**Trauma control**																						
Paper-and-pencil	1. OTBM	1																				
	2. GDS	−0.84^a^	1																			
	3. NDS-W	−0.94^a^	0.89^a^	1																		
	4. LSC	0.94^a^	−0.88^a^	−0.99^a^	1																	
	5. #≤ 5th %tile	−0.62^a^	0.77^a^	0.67^a^	−0.64^a^	1																
	6. #≤ 16th %tile	−0.86^a^	0.94^a^	0.89^a^	−0.89^a^	0.62^a^	1															
	7. #< 50th %tile	−0.91^a^	0.77^a^	0.92^a^	−0.91^a^	0.49^a^	0.82^a^	1														
CANTAB	8. OTBM	0.40^a^	−0.30^a^	−0.36^a^	0.37^a^	−0.36^a^	−0.29^b^	−0.38^a^	1													
	9. GDS	−0.37^a^	0.23^b^	0.32^a^	−0.34^a^	0.30^b^	0.26^b^	0.40^a^	−0.74^a^	1												
	10. NDS-W	−0.40^a^	0.29^b^	0.36^a^	−0.37^a^	0.37^a^	0.28^b^	0.38^a^	−0.93^a^	0.82^a^	1											
	11. LSC	0.40^a^	−0.28^b^	−0.36^a^	0.36^a^	−0.36^a^	−0.25^b^	−0.37^a^	0.93^a^	−0.82^a^	−0.99^a^	1										
	12. #≤ 5th %tile	−0.27^b^	0.14	0.24^b^	−0.26^b^	0.21	0.17	0.31^a^	−0.56^a^	0.84^a^	0.66^a^	−0.64^a^	1									
	13. #≤ 16th %tile	−0.39^a^	0.27^b^	0.37^a^	−0.39^a^	0.26^b^	0.30^b^	0.43^a^	−0.74^a^	0.89^a^	0.84^a^	−0.82^a^	0.67^a^	1								
	14. #< 50th %tile	−0.43^a^	0.38^a^	0.39^a^	−0.40^a^	0.46^a^	0.33^a^	0.37^a^	−0.91^a^	0.61^a^	0.89^a^	−0.89^a^	0.39^a^	0.63^a^	1							
Combined	15. OTBM	0.79^a^	−0.65^a^	−0.74^a^	0.74^a^	−0.55^a^	−0.64^a^	−0.73^a^	0.85^a^	−0.64^a^	−0.80^a^	0.80^a^	−0.48^a^	−0.65^a^	−0.80^a^	1						
	16. GDS	−0.75^a^	0.76^a^	0.77^a^	−0.77^a^	0.65^a^	0.74^a^	0.72^a^	−0.64^a^	0.74^a^	0.69^a^	−0.67^a^	0.61^a^	0.70^a^	0.60^a^	−0.82^a^	1					
	17. NDS-W	−0.79^a^	0.73^a^	0.81^a^	−0.81^a^	0.62^a^	0.73^a^	0.76^a^	−0.75^a^	0.68^a^	0.79^a^	−0.78^a^	0.54^a^	0.72^a^	0.73^a^	−0.91^a^	0.93^a^	1				
	18. LSC	0.81^a^	−0.73^a^	−0.82^a^	0.83^a^	−0.60^a^	−0.73^a^	−0.77^a^	0.75^a^	−0.67^a^	−0.78^a^	0.78^a^	−0.52^a^	−0.71^a^	−0.73^a^	0.93^a^	−0.92^a^	−0.99^a^	1			
	19. #≤ 5th %tile	−0.56^a^	0.57^a^	0.58^a^	−0.57^a^	0.75^a^	0.49^a^	0.51^a^	−0.60^a^	0.72^a^	0.67^a^	−0.66^a^	0.74^a^	0.59^a^	0.56^a^	−0.68^a^	0.85^a^	0.77^a^	−0.74^a^	1		
	20. #≤ 16th %tile	−0.76^a^	0.74^a^	0.78^a^	−0.80^a^	0.52^a^	0.80^a^	0.76^a^	−0.61^a^	0.69^a^	0.67^a^	−0.64^a^	0.51^a^	0.78^a^	0.56^a^	−0.80^a^	0.91^a^	0.92^a^	−0.91^a^	0.66^a^	1	
	21. #< 50th %tile	−0.76^a^	0.65^a^	0.75^a^	−0.75^a^	0.53^a^	0.65^a^	0.78^a^	−0.80^a^	0.59^a^	0.78^a^	−0.78^a^	0.41^a^	0.62^a^	0.85^a^	−0.94^a^	0.78^a^	0.89^a^	−0.90^a^	0.61^a^	0.77^a^	1
	22. Vocabulary	0.28^b^	−0.31^b^	−0.27^b^	0.28^b^	−0.24^b^	−0.25^b^	−0.27^b^	0.22	−0.09	−0.12	0.13	−0.03	−0.11	−0.14	0.30^b^	−0.21	−0.21	0.23	−0.19	−0.19	−0.23
**Community control**																						
Paper-and-pencil	1. OTBM	1																				
	2. GDS	−0.84^a^	1																			
	3. NDS-W	−0.95^a^	0.89^a^	1																		
	4. LSC	0.96^a^	−0.89^a^	−99^a^	1																	
	5. #≤ 5th %tile	−0.68^a^	0.85^a^	0.76^a^	−0.73^a^	1																
	6. #≤ 16th %tile	−0.86^a^	0.89^a^	0.90^a^	−0.90^a^	0.68^a^	1															
	7. #< 50th %tile	−0.92^a^	0.73^a^	0.89^a^	−0.90^a^	0.53^a^	0.81^a^	1														
CANTAB	8. OTBM	0.23	−0.15	−0.22	0.24^b^	−0.09	−0.24^b^	−0.31^a^	1													
	9. GDS	−0.18	0.11	0.17	−0.19	0.08	0.10	0.26^b^	−0.74^a^	1												
	10. NDS-W	−0.29^b^	0.19	0.26^b^	−0.28^b^	0.14	0.25^b^	0.34^a^	−0.91^a^	0.84^a^	1											
	11. LSC	0.29^b^	−0.21	−0.27^b^	0.29^b^	−0.15	−0.26^b^	−0.34^a^	0.92^a^	−0.84^a^	−0.99^a^	1										
	12. #≤ 5th %tile	−0.12	0.06	0.09	−0.12	0.05	0.03	0.19	−0.60^a^	0.82^a^	0.70^a^	−0.68^a^	1									
	13. #≤ 16th %tile	−0.18	0.11	0.17	−0.19	0.09	0.11	0.26^b^	−0.76^a^	0.96^a^	0.84^a^	−0.84^a^	0.74^a^	1								
	14. #< 50th %tile	−0.28^b^	0.17	0.25^b^	−0.27^b^	0.06	0.25^b^	0.35^a^	−0.90^a^	0.64^a^	0.90^a^	−0.91^a^	0.52^a^	0.67^a^	1							
Combined	15. OTBM	0.80^a^	−0.66^a^	−0.77^a^	0.79^a^	−0.52^a^	−0.72	−0.79^a^	0.74^a^	−0.55^a^	−0.73^a^	0.74^a^	−0.45^a^	−0.57^a^	−0.72^a^	1						
	16. GDS	−0.71^a^	0.77^a^	0.74^a^	−0.75^a^	0.69^a^	0.67^a^	0.67^a^	−0.53^a^	0.65^a^	0.63^a^	−0.64^a^	0.57^a^	0.61^a^	0.50^a^	−0.81	1					
	17. NDS-W	−0.77^a^	0.74^a^	0.81^a^	−0.82^a^	0.64^a^	0.74^a^	0.76^a^	−0.64^a^	0.60^a^	0.72^a^	−0.73^a^	0.52^a^	0.59^a^	0.64^a^	−0.91	0.93	1				
	18. LSC	0.78^a^	−0.72^a^	−0.81^a^	0.82^a^	−0.58^a^	−0.75^a^	−0.78^a^	0.67^a^	−0.61^a^	−0.74^a^	0.75^a^	−0.51^a^	−0.61^a^	−0.68^a^	0.93	−0.91	−0.99	1			
	19. #≤ 5th %tile	−0.58^a^	0.68^a^	0.63^a^	−0.62^a^	0.80^a^	0.52^a^	0.51^a^	−0.42^a^	0.54^a^	0.52^a^	−0.51^a^	0.60^a^	0.49^a^	0.36^a^	−0.66	0.89	0.81	−0.76	1		
	20. #≤ 16th %tile	−0.76^a^	0.71^a^	0.78^a^	−0.80^a^	0.54^a^	0.81^a^	0.78^a^	−0.64^a^	0.61^a^	0.68^a^	−0.69^a^	0.46^a^	0.64^a^	0.61^a^	−0.90	0.87	0.92	−0.94	0.69	1	
	21. #< 50th %tile	−0.72^a^	0.54^a^	0.69^a^	−0.71^a^	0.35^a^	0.64^a^	0.83^a^	−0.73^a^	0.54^a^	0.74^a^	−0.75^a^	0.43^a^	0.56^a^	0.81^a^	−0.92	0.71	0.84	−0.88	0.53	0.84	1
	22. Vocabulary	0.32^a^	−0.25^b^	−0.27^b^	0.30^b^	−0.12	−0.16	−0.29^b^	0.40^a^	−0.40^a^	−0.37^a^	0.38^a^	−0.33^a^	−0.46^a^	−0.34^a^	0.43^a^	−0.37^a^	−0.37^a^	0.41^a^	−0.27^b^	−0.37^a^	−0.39^a^

#, Number of scores; GDS, Global Deficit Score; LSC, Low Score Composite; NDS-W, Neuropsychological Deficit Score-Weighted; OTBM, Overall Test Battery Mean;

a p < 0.01;

b *p < 0.05*.

## Discussion

This study is part of a program of research designed to develop and evaluate a cognition endpoint suitable for TBI research and clinical trials (17, 19, 20). This study calculated and evaluated seven candidate composite scores for the neuropsychological battery used in the CENTER-TBI. The MTBI sample, on average, performed in the broadly normal range on all of the individual cognitive test scores at 2 weeks post injury (Table 2), which is generally consistent with studies examining neuropsychological outcome at 2 weeks after injury (13–16) and reflects the favorable cognitive outcome experienced by this sample. Further, there were no statistically significant differences in any of the composite scores, or in the individual tests, when comparing the MTBI, trauma control, and community control groups (Tables 2, 7). Within the MTBI group, subgroups were created based on injury severity. There were no statistically significant differences on any composite when comparing those with complicated vs. uncomplicated MTBI (Table 7), which is somewhat surprising given that participants were evaluated only 2 weeks following injury. However, the literature on neuropsychological outcome from complicated MTBI during the acute and subacute period following injury is mixed, with some studies showing greater cognitive deficits and some studies showing comparable cognitive functioning to those with uncomplicated MTBIs (39–48). The majority of the patients were discharged to home from the emergency department. Thus, our sample represents the milder end of MTBI, possibly contributing to the lack of differences between complicated and uncomplicated MTBI. There were no statistically significant differences on any composite when comparing participants with and without LOC at the time of injury (Table 7). There were significant differences on multiple composites between those with a greater duration of PTA at the time of injury and those with a shorter duration of PTA (Table 7), a finding that aligns with some previous research (49, 50). However, these results are confounded by participants with longer duration of PTA having lower estimated longstanding intellectual functioning than those with short duration PTA. Because intellectual functioning is known to be correlated with neuropsychological test performance (51–53), and with the composite scores in the present study (Tables 8, 9), the findings are likely in part related to premorbid differences in intelligence.

Two of the composite scores had distributional characteristics that were approximately normal (i.e., the OTBM and the #< 50th %tile composites; Tables 3, 4). Most of the composite scores, however, had skewed distributions because they are based on deficit scores, or the number of low scores. Some have notable zero-inflation, meaning that a large number of people obtain a score of zero (Tables 3, 4). The distributional characteristics of the composite scores are important to consider when choosing a composite score for TBI research and clinical trials because these characteristics have consequences for the statistical methods available for data analyses. There is often a need to control for demographic variables, such as education or intelligence, or injury severity variables, in the statistical analyses in TBI research. The OTBM score will approach a normal distribution in most cases and covariates can easily be included in traditional parametric methods, such as ordinary least squares regression. The zero-inflated composites, however, likely require non-parametric methods (e.g., Mann-Whitney *U*-tests used in the present study), which do not allow for covariates. If there is a need to include covariates, regression models developed for count data (e.g., Poisson or negative binominal regression) could be an alternative for the zero-inflated composite scores.

Intercorrelations between the composite scores were higher *within* a test battery (paper-and-pencil; CANTAB; combined) than *between* one test battery and a different test battery (Tables 8, 9). The correlation of 0.55 between the paper-and-pencil OTBM composite and the CANTAB OTBM composite in the MTBI group is not surprisingly low considering that in concurrent validity studies on neuropsychological tests, it is common to find only moderate correlations between tests designed to measure even the same cognitive function (54, 55). Moreover, the correlation might be somewhat attenuated due to method variance (i.e., computerized vs. traditional testing). Despite a moderate correlation, the effect sizes were broadly similar for the paper-and-pencil battery and the CANTAB battery when groups and subgroups were compared. Based on this, we cannot conclude that one of the batteries (paper-and-pencil and CANTAB) is more sensitive than the other. Within the paper-and-pencil composites and the CANTAB composites, the NDS-W and LSC were extremely highly correlated (i.e., 0.99 in almost all groups and all batteries), which is consistent with previous findings (19, 20). It is likely that these two composites are redundant. The OTBM and the #< 50th %tile composites were also highly correlated. Correlations above 0.90 between these composites have also been found in the D-KEFS battery (20) and in the ANAM4 TBI-MIL battery (19). Therefore, researchers interested in using more than one of these composite scores are encouraged to avoid including redundant scores as outcome measures and instead choose two composites with lower intercorrelation (such as the OTBM and the #≤ 5th %tile).

The present study differs from previous cognition endpoint research (19, 20) in that the tests in the neuropsychological battery used were not co-normed. Thus, when the age-referenced T scores were calculated (Table 2), different norm groups were used for the different neuropsychological tests. In both TBI research and neuropsychological clinical practice, this is a very common scenario. There are dozens of tests recommended as common data elements outcome measures following TBI (56), and these tests have diverse sets of normative reference values. Using cognitive tests from multiple normative samples, as we did in this study, mirrors typical research and clinical practice and increases the generalizability of these findings. However, the use of different norms requires some extra considerations. Even though T scores are designed so that the mean is 50 and the standard deviation is 10 in the normative sample, this is not necessarily the case in the groups of interest in a study. Obtained scores in a study sample often vary from that expected distribution due to characteristics of the sample (e.g., level of education or intellectual functioning), differences in the normative reference groups, or both. For example, in the community control group in present study, PAL scores derived from the CANTAB normative sample had a mean of 51.80 and a standard deviation of 6.55, while RAVLT Trial 1–5 scores derived from the Schmidt et al. meta-norms (30) had a mean of 48.19 and a standard deviation of 11.60 (Table 2). Assuming a normal distribution of the scores in the community control group, this means that a score at the 16th percentile (e.g., one standard deviation below the mean for the community control group) for the PAL would be a norm-referenced T score of about 45 and a score at the 16th percentile on the RAVLT would be a norm-referenced T score of about 37. Consequently, observed deficit scores would vary by individual measures. For example, fewer participants would have T scores low enough to lead to a positive GDS on the PAL than the RAVLT, because the GDS requires the score to be lower than a norm-referenced T score of 40 to be a non-zero value. Although a similar portion of the community control group (~16%) obtained a T score ≤45 on the PAL and ≤37 on the RAVLT, the influence these scores have on the GDS would not be equivalent. It should be noted that the same situation also can arise from batteries that are co-normed (i.e., the standard deviations will, more or less, deviate from 10 in the study groups), but that differences may be more pronounced when different norms are used.

Prior developmental work on this topic has been done exclusively with people with MTBIs or with healthy participants (19, 20). Although this study extends prior work by having MTBI participants, trauma controls, and community controls in the same study, there is a need to compare and contrast these composite scores in people who have sustained moderate or severe TBIs and who have varying degrees of cognitive impairments before determining which cognition endpoint is most useful for TBI research and clinical trials. An additional limitation of the present study relates to the representativeness of the norms used for composite score calculation. The sample was recruited from Norway and the tests were administered in Norwegian, but none of the normative samples were comprised of people from Norway. This is the clinical and research reality in many countries; normative samples for many neuropsychological tests are from North America, mostly from the United States. The comparisons in the present study, however, were between groups, all of which were assessed with the same tests and norms, making the representativeness of the norms less confounding. Further, the mean OTBM for the community controls was 51.21 (i.e., close to the norm group mean of 50), indicating the mean performances for the observed sample were similar to those of the normative samples. The subgroup comparisons in the present study are limited by the relatively small number of patients with complicated MTBI (*n* = 17) and patients without LOC (*n* = 26). Because we required LOC to be witnessed in order to be coded as present, many patients were excluded from the LOC vs. no LOC comparisons due to uncertain LOC status. Thus, the lack of differences between patients with complicated and uncomplicated MTBI, and between patients with and without LOC, should be interpreted with caution. Also, the duration of PTA was self-reported and there is some evidence that people tend to overestimate their PTA duration, especially patients with cognitive deficits (57). The finding of lower cognitive functioning in patients with longer PTA could have been influenced by reporting bias. Finally, no stand-alone performance validity tests were included in this study. The context in which this study took place may make this concern less critical, but still important. We recruited a representative sample of MTBI patients in the acute phase, and not patients seeking health care later and test results from this study were solely for research purposes, not available to anyone else, and no financial gain could be obtained.

This study adds to a body of literature exploring methods to aggregate neuropsychological test scores in a manner that could be useful to produce summary outcome scores for use in observational research and clinical trials. It is an explicit goal of this program of research to develop a cognition endpoint score suitable for diverse batteries of tests, administered in different countries and in different languages, to people with TBIs of broad severity and at varying time points following injury (17). In the present study, cross-sectional comparisons between MTBI, trauma control, and community control groups 2 weeks following injury showed that the seven composites had similar effect sizes of group differences, along with significant redundancy between some of the composite scores as evidenced by high intercorrelations. If future studies on moderate to severe TBI confirm similar effect sizes between the composites, the OTBM composite may be preferred as a clinical trial endpoint because no data reduction (i.e., using cutoffs for defining a low score that are then changed into specific values) is needed when this composite is calculated. Also, the risk of unequal impact from different scores when several normative samples are used might be smaller for the OTBM score because this composite does not rely on cutoffs based on normative data. For studies seeking to enrich or stratify, however, a composite score that classifies people as having mild cognitive impairment might be preferred. The studies to date have examined the distributional characteristics of composites, similarities between them, and group differences based on mild injuries. These studies have had largely similar findings. Future studies examining additional psychometric aspects of the composite scores (e.g., test-retest reliability, reliable change, and minimal clinically important differences) are needed.

## Data Availability Statement

The datasets generated for this study are available on request to the corresponding author.

## Ethics Statement

The studies involving human participants were reviewed and approved by the regional committee for research ethics (REK 2013/754). The patients/participants provided their written informed consent to participate in this study.

## Author Contributions

JS, JK, DT, NS, and GI designed the study. JS performed the statistical analyses. TS and AV secured funding for the larger parent study and directed that study. JS, SS, and TS collected the data. JS and GI drafted the manuscript. All authors critically reviewed the manuscript, read, and approved the last version of this manuscript.

## Conflict of Interest

GI serves as a scientific advisor for BioDirection, Inc., Sway Operations, LLC, and Highmark, Inc. He has a clinical and consulting practice in forensic neuropsychology, including expert testimony, involving individuals who have sustained mild TBIs. He has received research funding from several test publishing companies, including ImPACT Applications, Inc., CNS Vital Signs, and Psychological Assessment Resources (PAR, Inc.). He received royalties from one neuropsychological test (WCST-64; PAR, Inc.). He has received research funding as a principal investigator from the National Football League, and salary support as a collaborator from the Harvard Integrated Program to Protect and Improve the Health of National Football League Players Association Members. He acknowledges unrestricted philanthropic support from ImPACT Applications, Inc., the Heinz Family Foundation, and the Mooney-Reed Charitable Foundation. The remaining authors declare that the research was conducted in the absence of any commercial or financial relationships that could be construed as a potential conflict of interest.
